# Corticomuscular Coherence and Its Applications: A Review

**DOI:** 10.3389/fnhum.2019.00100

**Published:** 2019-03-20

**Authors:** Jinbiao Liu, Yixuan Sheng, Honghai Liu

**Affiliations:** State Key Laboratory of Mechanical System and Vibration, School of Mechanical Engineering, Shanghai Jiao Tong University, Shanghai, China

**Keywords:** corticomuscular coherence, magnetoencephalography, electroencephalogram, surface electromyogram, stroke

## Abstract

Corticomuscular coherence (CMC) is an index utilized to indicate coherence between brain motor cortex and associated body muscles, conventionally. As an index of functional connections between the cortex and muscles, CMC research is the focus of neurophysiology in recent years. Although CMC has been extensively studied in healthy subjects and sports disorders, the purpose of its applications is still ambiguous, and the magnitude of CMC varies among individuals. Here, we aim to investigate factors that modulate the variation of CMC amplitude and compare significant CMC between these factors to find a well-developed research prospect. In the present review, we discuss the mechanism of CMC and propose a general definition of CMC. Factors affecting CMC are also summarized as follows: experimental design, band frequencies and force levels, age correlation, and difference between healthy controls and patients. In addition, we provide a detailed overview of the current CMC applications for various motor disorders. Further recognition of the factors affecting CMC amplitude can clarify the physiological mechanism and is beneficial to the implementation of CMC clinical methods.

## Introduction

Preview human studies used positron emission tomography (PET), functional magnetic resonance imaging (fMRI), transcranial magnetic stimulation (TMS) or electroencephalogram (EEG) to suggest the underlying mechanisms of motor cortex in patients with strokes (Mima et al., 2001; Zheng et al., 2017). However, the exact role of how ipsilateral motor cortex or secondary motor areas control the muscle activity is still to be fully discovered. One approach to overcome this issue is to measure EEG signals and corresponding EMG signals simultaneously. This method is known as corticomuscular coherence, which is considered to be a classic and commonly used approach to assess the synchrony between neural signals and associated body muscles. Corticomuscular coherence (CMC) was initially reported between magnetoencephalography (MEG) and electromyography (EMG) (Kilner et al., 2000; Tecchio et al., 2006, 2008; Porcaro et al., 2008) and is widely detected by techniques such as EEG, electrocorticography (ECoG), surface electromyography (sEMG), and has thus been validated across methods and species (Gerloff et al., 2006).

**FIGURE 1:**
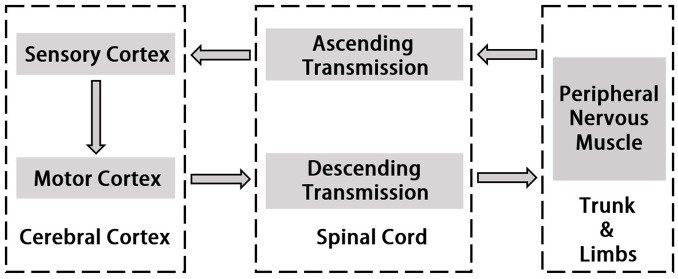
Schematic diagram of signal transmission in cerebral cortex and peripheral nerve.

Corticomuscular coherence is a common and useful method to study the mechanism of cerebral cortex’s control of muscle activity. It reveals functional connection between the cortex and muscles during continuous muscle contractions. The origin of CMC is the communication in corticospinal pathways between primary motor cortex and muscles. Normally, cortical events propagate to the periphery and motor cortex also receives input from the periphery (Salenius et al., 1997; Gross et al., 2000; Riddle and Baker, 2005). Horak’s motion control theory emphasizes “Normal motion control refers to the central nervous system by using existing and past information to transform neural energy into kinetic energy and enable it to perform effectively functional activities” (Horak, 1991). In this process, the interaction between the two systems of central nervous system and motor muscle tissue is included. Utilizing hand grip as an example, the command which is issued by the motor cortex will be carried down along the motor conduction pathway and dominates upper body’s peripheral nerves and muscles when motion occurs (Claudio et al., 2008). The sense of proprioception is simultaneously conducted along the sensory conduction pathway to the spinal cord, the brain stem and the cerebellum, and partly to the cerebral hemisphere. Most of the proprioceptive information are transmitted to the sensory regions of the brain for comprehensive analysis and regulate motion commands (Witham et al., 2011). The study of the cortical-muscle function coupling can reflect the interaction between the cerebral cortex and the muscle tissue which represents the flow of information within the motion system and is associated with the cerebral cortex sending commands to the muscle tissue and the afferent feedback of muscle contraction. Thus, it serves to understand how the brain controls muscle tissue, the effects of muscle movement on brain function and the explanations of the rooted causes of specific physiological conditions such as fatigue. More recent studies using directional coherence analysis have emphasized that CMC reflects both the corticoefferent descending locomote from motor cortex to muscles as well as ascending corticoafferent locomote from muscles to motor cortex in producing the CMC (Hellwig et al., 2001; Fang et al., 2009; Airaksinen et al., 2015a). Figure 1 indicates the pathway of signal transmission between cortex and muscle.

Since Conway et al. (1995) reported increased coherence between MEG signals in the contralateral motor areas and the surface EMG (sEMG) signal during muscle contractions, CMC has been extensively studied for the continuous contraction of limb muscles (Kilner et al., 2000; Krause et al., 2013; Rossiter et al., 2013; Maezawa, 2016; Matsuya et al., 2017). Based on previous studies, it can be suggested that the magnitude of CMC reflects the indicator of human neurophysiology in both healthy subjects and sport disorders. However, neural system is extremely complex and diverse among individuals, which causes the CMC amplitude would be emerged different correlation results under different research conditions. Hellwig et al. (2001) applied EEG to reveal tremor-correlated cortical activity in tremor patients. EMG signals of wrist extensor and flexor muscles were recorded from tremor side of patients. With EEG recording, CMC was estimated and found that there was a highly significant coherence at the tremor frequency. Raethjen et al. (2002) discovered that CMC had a significant coherence in the 6–15 Hz range in four out of the six tremor patients. That because corticomuscular transmission of the oscillation was in progress between cortex and muscles rather than peripheral feedback to the cortex. This research pointed out that the band frequency was an important factor which may impact CMC. To compare CMC between young and older adults, Johnson and Shinohara (2012) discussed CMC and fine motor performance during the unilateral fine motor task and concurrent motor and cognitive tasks which asked participants to increase the force from zero to maximum using the index finger. From this study, results revealed that older adults had lower CMC in beta-band and higher alpha-band than young adults during dual tasks and young controls, rather than older adults, with greater beta-band CMC exhibited accurately motor output. Previous researches have confirmed that CMC magnitude often varies greatly due to different experimental designs, magnitude of exerted force, or individual differences. However, there are no relevant studies to make a detailed investigation of the factors affecting the amplitude of CMC.

Compare with the coherence between all coupling degrees, the classifications which have significant coherence could be observed. These can serve as the research emphasis in the future. The remaining of this review is organized as follows: section *CMC DEFINITION AND FORMULAE* proposes a generalized definition of CMC; section *FACTORS AFFECTING CMC* provides a detailed overview of the factors affecting the corticomuscular coordination, including experimental design (von Carlowitz-Ghori et al., 2015), band frequencies (Schulz et al., 2014; Maezawa et al., 2016) and force levels (Dal Maso et al., 2017), age correlation (Kamp et al., 2013) and difference between healthy controls and patients (Krause et al., 2013; Rossiter et al., 2013); the applications of CMC for patients with various motor disorders are further presented in section *CMC APPLICATIONS*; section *DISCUSSION* proposes a discussion of CMC modulation and future directions; the paper is concluded in section *CONCLUSION*.

## CMC Definition and Formulae

Coherence is an indicator of the linear connection between two signals (Grosse et al., 2003) and is an extension of Pearson correlation coefficient in the frequency domain (Mima and Hallett, 1999). Coherence was obtained from the normalization of the cross-spectrum (Fang et al., 2009):

$${Coh}_{S1,S2}(f)=\frac{{|P_{S1,S2}\left(f)\right|}^{2}}{|P_{S1}(f)|\times |P_{S2}\left(f)\right|}$$

$$P_{S1,S2}(f)=\frac{1}{n}\overset{n}{\underset{i=1}{\Sigma }}{S1}_{i}(f){S2*}_{i}\left(f\right)$$

Where *P*_*S*1,*S2*_ (*f*) is the cross-spectrum density of the signal, *P*_*S*1_ (*f*) and *P*_*S*2_ (*f*) are the auto-spectrum densities of signals *S*1 and *S*2, respectively, at frequency *f*. Values of coherence is normalized and will always satisfy 0 to 1 where 1 indicates an ideal correlation between two signals and 0 indicates a total absence of association.

Corticomuscular coherence is an implement to understand how cortical activities control the muscle movements and examines the functional coupling between brain motor cortex and associated muscles. Ascending and descending corticomuscular pathways are two diverse directions which could both generate coherence, however, descending pathway are more clearly and certainly than ascending pathway. Hence, the common definition of CMC indicates the cortex-muscle coherence underlying descending pathway.

As the variety of signal collection techniques, CMC shows a widely research space to analyze different types of signals collecting from different approaches. From the recent studies, the most familiar techniques to collect brain activity signals are EEG, MEG, and ECoG and muscle activity signals are sEMG and ultrasound. In the researches of CMC, EEG-EMG, MEG-EMG, and ECoG-EMG are the most three commonly used methods to analyze the functional coupling between brain cortex and muscle activities and these three sets of signals are used to calculate the coherence parameter. Therefore, in the equations (1) and (2), two signals *S*1 and *S*2 could represent these three types of signal combinations. At the same time, ultrasound is an unusual signal format to use in CMC analysis, which could be regarded as a future research field.

Equation (1) and equation (2) offer a basic and intuitive technique to display the synchronous values. On this basis, wavelet-based coherence is proposed to enhance the relative level of the motor cortex and muscle and observes the coherence in time-frequency domain which estimates the signal spectral characteristics according to the function of time (Xu et al., 2015). To overcome the problems of non-stationary signals like sEMG signal, wavelet analysis is a rational method to analyze signals with fast-changing spectra (Lachaux et al., 2002). One primary advantage of wavelet analysis is to observe the significant coherence in different time for different tasks intuitively and expediently. Compare to traditional CMC analysis result, wavelet coherence increases precision when analyzing temporary activities between two oscillatory neural signals and is good at dynamic neural interactions.

Morlet wavelet family is a simple and suitable wavelet for spectral estimations although there are still many wavelets could be chosen. The signal *x* (*u*) is decomposed along Morlet wavelet and under the frequency *f* and time τ, it could be calculated by the following formulae:

$$\psi_{\tau ,f}(u)=\sqrt{f}\cdot \exp(i2\pi f\left(u-\tau \right))\cdot \exp\left(-\frac{{\left(u-\tau \right)}^{2}}{\sigma^{2}}\right)$$

Where ψ_τ,*f*_ (*u*) is the product of a sinusoidal wave at frequency *f*, with a Gaussian function centered at time τ with a standard deviation σ proportional to the inverse of frequency *f*. The wavelet transform function *W_X_* (τ,*f*) of a signal *x* (*u*) is a function given by the convolution of *x* with Morlet wavelet family:

$$W_{X}(\tau ,f)=\int_{\left(-\infty \right)}^{\left(+\infty \right)}x(u)\cdot \psi_{\tau ,f}*(u)du$$

From the wavelet transform function, the wavelet cross-spectrum of two signals between brain cortex and muscle is as follow:

$${SW}_{S1,S2}(t,f)=\overset{t+\delta /2}{\underset{t-\delta /2}{\int }}W_{S1}(\tau ,f)\cdot W_{S2}*\left(\tau ,f\right)d\tau$$

Where δ is a scalar that can depend on frequency. Therefore, the wavelet coherence W*Coh*_*S*1,*S*2_ (*f*) could be defined as follow:

$${WCoh}_{S1,S2}(f)=\frac{|{{SW}_{S1,S2}\left(f)\right|}^{2}}{|{SW}_{S1,S1}(f)|\times |{SW}_{S2,S2}\left(f)\right|}$$

Besides the above two methods to analyze CMC, Fourier coherence and partial directed coherence are also reported in some researches (Lachaux et al., 2002; Porcaro et al., 2009; Mcmanus et al., 2013). Compare Fourier coherence with Wavelet coherence, these two methods both study non-stationary signals, however, the window size of wavelet analysis is fixed and it is more adapted to the frequency of the oscillatory signals. As a result, wavelet coherence has a more accurate consequence than Fourier coherence. For partial directed coherence, this technique could evaluate the flowing direction of neural information and indicate how cortical signals and muscular signals are functionally connected compared with ordinary CMC analysis. And this is a potential technique because the current researches are most focus on the synchrony between two signals or the descending corticospinal pathway.

With the intensive study of CMC, related analytical methods also evolve gradually. Researchers are not satisfied with commonly CMC analysis method; thus, Wavelet coherence has become a widely used technology in the recent years. Wavelet coherence could show CMC magnitude during the entire task time series. Since the strong coherence under specific movements could be observed, the discovery of the factors affecting CMC will be easier to achieve.

## Factors Affecting CMC

Research shows strong correlated area of CMC has been confirmed by direct electrical stimulation in monkeys and human surgery, including the anterior motor area, the primary motor area, the primary somatosensory area, the thalamus, the nucleus of the hypothalamus, and the cerebellum (Salenius and Hari, 2003), Figure 2 shows primary brain regions which related to CMC. However, several studies suggest that the magnitude of CMC is not only related to the corresponding regions of the cerebral cortex, but also directly related to the CMC band (Cottone et al., 2017; Porcaro et al., 2018). Chakarov et al. (2009) found that the CMC value of the 15–45 Hz frequency band increased linearly with the rise of the dynamic force level, that is, the CMC value was a high-level dynamic synchronization process. Survey also shows that the CMC value of the beta band (13–30 Hz) is related to the output of the static force, and the coherence of the gamma band (31–45 Hz) is related to the output of the dynamic force (Gwin and Ferris, 2012). The CMC magnitude is significantly lower in the case of unpredictable low-level force frequency (Mendez-Balbuena et al., 2013). The unpredictability of the force frequency could lead to the decrease of the corticospinal tract synchronism, the increase of cortical and muscle activation, and the decrease of motor performance. Several studies have demonstrated that beta-band CMC value is modulated by afferent information (Fisher et al., 2002; Pohja et al., 2002; Kilner et al., 2004; Riddle and Baker, 2005; Baker et al., 2006), and visuomotor tasks (Perez et al., 2006).

**FIGURE 2:**
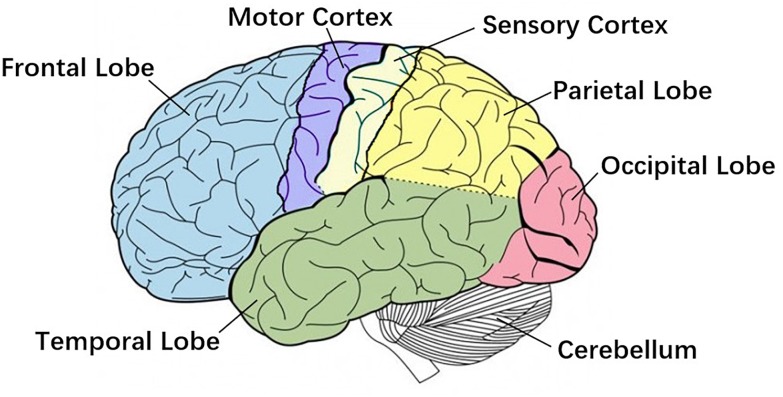
Primary brain regions. Motor cortex is the region in charge of planning, control and execution of voluntary movements. Sensory cortex arranges tactile representation from the toe to mouth. Cerebellum is mainly responsible for motion control. These three regions are more related to Corticomuscular coherence (CMC).

Moreover, the CMC value of healthy subjects is generally higher than that of sport disorders. Stroke patients have a significantly lower corticomuscular coherence compare with healthy controls at both the beta (20–30 Hz) and lower gamma (30–40 Hz) bands during the movement (Fang et al., 2009). During light voluntary muscular contraction, beta-band CMC is markedly reduced in Amyotrophic Lateral Sclerosis patients compare with healthy controls (Proudfoot et al., 2018). However, research also indicates that stroke survivors manifest a more distributed range of cortical locations for peak CMC than healthy controls, in keeping with plastic reorganization of sensorimotor functionality (Farmer et al., 1993; Rossiter et al., 2013). In addition, with the rehabilitation of motor function, the value of CMC will increase gradually on sport disorder patients. Motor deficits secondary to acute stroke are accompanied by a unilateral reduction in CMC, which then normalizes with good functional recovery (von Carlowitz-Ghori et al., 2014).

Although the factors that affect the result of CMC amplitude have not been specifically counted, the classification and comparison of the current research focus on CMC can provide a more specific understanding of the mechanism of CMC. The factors affecting the corticomuscular coordination are summarized as follows: experimental design, band frequencies and force levels, age correlation and difference between healthy controls and patients.

### Experimental Design

The magnitude of CMC is closely related to the paradigm design. Different experimental paradigms may result in separate CMC magnitude. Table 1 displays different CMC experimental design. Force is one of the most significant indices in CMC experiment generally which is relevant to the form of muscle contraction during the experimental design, such as isometric contraction, isokinetic contraction, isotonic contraction, etc. Most studies used isometric contraction as a form of muscle contraction in CMC experiments. Dal Maso et al. (2017) investigated that whether CMC magnitude differed with torque levels during isometric knee contractions tasks. The net joint torque, muscles co-activation and CMC values were comparable when participants performed complete isometric elbow flexion exercise with three force output levels (Cremoux et al., 2017). Similarly, in order to explore whether oscillatory activity could contribute more to the stability of isometric muscle contraction, the subjects were required to perform steady isometric contractions, using two different finger muscles (Lim et al., 2014). Current studies on isokinetic and isotonic contractions are relatively limited. Intramuscular Tibialis Anterior (TA) coherence estimation was investigated within a specific frequency range during 120° isokinetic movement (Bravo-Esteban et al., 2014), and Yang et al. (2016) proposed a measure for evaluating non-linear corticomuscular coupling during isotonic wrist flexion.

**Table 1:** Different experimental design of CMC.

Reference	Contraction form	Muscle position	Sample	CMC results
Dal Maso et al., 2017	Isometric	Agonist Antagonist	21 right-footed men	CMC magnitude decreased more in antagonist than in agonist muscles as torque level increased.
Cremoux et al., 2017	Isometric	Antagonist	8 SCI patients 10 healthy participants	Magnitude of CMC and muscle co-activation decreased with the increase in the force level.
Lim et al., 2014	Isometric	FPB FDMB	15 healthy subjects	Greater β-band DTF was associated with high EMG stability levels and greater β-band CMC strength.
Matsuya et al., 2017	Isometric	FDI SOL	16 healthy young adults	A significant, positive correlation between recurrent inhibition and peak CMC across individuals.
Rossiter et al., 2013	Isometric	Forearm flexors and extensors	25 stroke patients23 healthy controls	Peak CMC in the contralesional hemisphere was found not only in some highly impaired patients, but also in some patients with good functional recovery.
Rong et al., 2014	Isometric	Right EDC	27 healthy subjects	CMC might represent a general marker of aging increased coherence amplitude might denote a compensatory mechanism to maintain isometric contraction.
Bravo-Esteban et al., 2014	Isokinetic	TA	14 SCI subjects15 healthy controls	Analysis of intramuscular TA coherence during isometric activation is related to muscle strength and gait function following incomplete SCI.
Yang et al., 2016	Isotonic	FCR	11 healthy subjects	The corticospinal tracks mainly mediate linear corticomuscular coupling, while non-linear coupling might relate to sensory feedback pathways.


*SCI, spinal cord injury; FPB, flexor pollicis brevis, FDMB, flexor digiti minimi brevis; FDI, first dorsal interosseous; SOL, soleus; EDC, extensor digitorum communis; TA, tibialis anterior; FCR, flexor carpi radialis.*

Muscle fatigue is an unavoidable problem in the CMC experiment, therefore the time design of the experiment is an essential part, including the duration of the continuous force, the rest time, etc. For example, when subjects performed two specific tasks according to the prompts on the monitor, they needed to rest for 10 min between each trial to prevent muscle fatigue (Rong et al., 2014). Besides, a study of whether CMC magnitude in beta-band differed with torque levels required subjects to perform three 4s knee isometric MVC (maximum voluntary contraction) and 6s rMVC (relative maximum voluntary contraction) in both directions of contraction (Dal Maso et al., 2017). For experimental design with patient’s participation, such as a stroke patient’s experiment, the design of time interval between two experiments are required to ensure that the patient’s affected side has achieved significant motor function recovery (Zheng et al., 2017).

Localization of muscle position by EMG electrodes is the underlying cause of CMC amplitude variation. From previous studies, normally, the acquisition of muscle signals derives from the limbs and hands. For instance, Lou et al. (2013) executed 4 hand movement tasks to investigate CMC. Rong et al. (2014) proved sensorimotor cortex enhanced communication with the measured muscle in right hand. EMG of biceps brachii muscle had also been studied to analyze the effects of mechanically amplified tremor on CMC (Budini et al., 2014). Otherwise, some studies have evaluated the influence of stance width, vision, and surface compliance on beta CMC during human stance. The results showed that under the condition of wide-stance, CMC amplitude is obviously larger than that under the condition of narrow-stance (Jacobs et al., 2015). However, no study placed electrodes in human trunk, the reason for this result is probably that the muscle contraction degree in human trunk is considerably lower than that in the limbs, and the EMG signal obtained is not enough to achieve obvious CMC amplitude.

Moreover, some comparative studies have demonstrated the effects of different forms of tasks in CMC. For example, study found that functional coupling between cortex activity and muscles was less in position-control task than in force-control task (Poortvliet et al., 2015). Similarly, research indicated that motor control strategies differed between force and position control tasks (Maluf and Enoka, 2005). Comparing to the position control task, EEG power of beta-range in the force control task showed greater activity desynchronization (Pfurtscheller and Lopes da Silva, 1999). For patients, the comparison of the CMC task between the affected side and the unaffected side is usually adopted. The study found that the frequency of CMC on the affected side reduced and the magnitude of CMC on the unaffected side increased in acute stroke (von Carlowitz-Ghori et al., 2014).

Experimental design is the initial step of studying CMC. Only a scientific and reasonable paradigm is possible to achieve satisfactory results. Future research on CMC experimental design should be transferred to isokinetic contraction and isotonic contraction since isokinetic movement excludes the muscle force is different during dynamic muscle contraction among individual which is superior to isometric contraction. Time design requires full consideration of the characteristics of muscle fatigue of diverse subjects and more reasonable allocation. Meanwhile, changes of muscle activity in human trunk are also the direction of future CMC research.

### Band Frequencies and Force Levels

The corticomuscular coherence at a certain frequency is a function of power spectral density (PSD) and cross-spectral density (CSD), which indicates that frequency bands affect the CMC amplitude. Oscillations in the beta-range (14–30 Hz) are explicitly observed in recording EEG from the cerebral motor cortex (Budini et al., 2014). Significant coherence of beta-range between sensorimotor cortex and contraction muscles has been reported for the first time. Significant beta band coherent activities between the sensorimotor cortex and contracting muscle were proposed in both monkeys (Baker et al., 1997) and humans (Conway et al., 1995) around 20 years ago. Similar oscillations can also be observed in the EMG of forearm and medial muscles of hand during sustained contraction (Conway et al., 1995; Baker et al., 2003). More prominent beta-range rhythmic EMG burst accompanies with higher CMC. Ushiyama et al. (2011a) described that the amplitude of CMC was positively correlated with the beta-range oscillation of EMG signal. Besides, the experimental data also showed that there was a significant correlation between the CMC amplitude and the beta-range intensity of EMG. CMC was noteworthy in both 13–21 and 21–31 Hz frequency bands in flexors and extensors regardless of subject group, torque level or direction of contraction (Dal Maso et al., 2017). In addition, research on alpha-band (8–13 Hz) and gamma (30–80 Hz) was also being undertaken. Alpha-range coherence showed advanced EMG reflecting ascending or feedback interactions and gamma-range coherence revealed delayed EMG activity indicated descending or feedforward interactions (Mehrkanoon et al., 2014).

Studies in participants with Parkinson’s disease and essential tremor, however, have observed significant coherence between cerebral cortex and peripheral EMG activities in the alpha-range and the frequency range of pathological tremor (4–6 Hz) (Hellwig et al., 2000; Timmermann et al., 2003; Raethjen et al., 2007). In addition, significant peak CMC has been revealed at 8–12 Hz when healthy subjects imitated Parkinsonian resting tremor at 3–6 Hz (Pollok et al., 2004). From the present study, it has been proved that beta band is the focus of CMC research. CMC has stronger volatility and more obvious amplitude in this frequency band. However, the CMC amplitude of sport disorder patients at lower frequencies are easy to be observed.

In current CMC researches, diverse force levels are normally studied along with different band frequencies. Table 2 shows some researches concerning force and bands. The muscular force level and the movement type can affect the CMC amplitude (Lattari et al., 2010). It has been further shown that the level of CMC increases with the strengthen EMG in healthy individuals (Kilner et al., 2000), which indicates that muscle output is dependent on CMC intensity. For example, to investigate the correlation of CMC in different MVC (maximum voluntary contraction) levels in both static and dynamic task of hand movement, the participants should reach 4, 8, and 16% MVC and the result showed that the amplitude of CMC tended to increase with the force increasing in static task and dynamic finger moving task and the CMC mainly concentrated in beta band (Fu et al., 2014). In the same way, Witte et al. (2007) also demonstrated a significant increase in CMC values of beta-range from 4 to 16% MVC which was associated with better performance. Thus, it could be seen that the force input level is closely related to the CMC frequency band, and prior studies have indicated that steady force is accompanied by beta-range CMC (Pfurtscheller and Neuper, 1992; Baker et al., 1997; Halliday et al., 1998; Kilner et al., 1999; Feige et al., 2000; Mima et al., 2000; Fu et al., 2014). Besides, studies demonstrated that CMC within the gamma band can be observed (Brown et al., 1998) during slow movements (Mima et al., 1999) and phasic movements for Previous studies also observed that gamma-range CMC has been related with isometric compensation of low dynamic force (4% MVC) and a markedly broad-band CMC (15–45 Hz) which composed of beta- and gamma- range was associated with the force level (Chakarov et al., 2009). These results show that the function of beta-range CMC is not limited to low-level steady forces. In addition, the sensorimotor system may resort to higher and also extended frequency range of CMC would generate stable corticospinal interaction during rising force standard (Lattari et al., 2010).

**Table 2:** Diverse force level and bands of CMC.

Reference	Force level	Bands	Sample	Significant CMC
Hori et al., 2013	1.96 N–3.92 N	Alpha/Theta	9 healthy subjects	Yes
Budini et al., 2014	20% MVC	Alpha	13 healthy subjects	Partially
Lim et al., 2014	20% MVC	Beta	15 healthy subjects	Yes
Ushiyama et al., 2017	30% MVC	Beta	22 healthy subjects	Yes
Mehrkanoon et al., 2014	target 1: 0.5–0.9 N target 2: 1.1–1.5 N	Alpha/Gamma	12 healthy subjects	No
Dal Maso et al., 2017	20,40, 60, and 80% of rMVC	Beta	10 ST subjects 11 ET subjects	CMC decreased
Rong et al., 2014	25 % MGF and 75 % MGF	Alpha/Beta/Gamma	14 healthy subjects	Alpha/Beta increased Gamma decreased
Fu et al., 2014	4, 8, and 16% MVC	Beta	8 healthy subjects	Yes


*MVC, maximum voluntary contractions; ST, strength-trained; ET, endurance-trained subjects; rMVC, relative MVC; MGF, maximum grip force.*

Furthermore, Lattari et al. (2010) also showed that greater corrective movements in the 4% MVC condition might reduce CMC. In line with that, the findings of Andrykiewicz et al. (2007) demonstrated that the amplitude of dynamic force did not modulate the gamma-range CMC, which suggested that changes in proprioceptive input during dynamic forces in the range from 1.6 to 4% MVC were insufficient for this modulation. In view of this, there is an explanation that weakening of cortical-muscular coupling may be the main neural mechanism induce to muscle fatigue and associate with performance impairment (Yang et al., 2009). Rong et al. (2014) found that when grip force increased, the sensorimotor cortex reduced communication in gamma band to keep stabilization. Besides, there are also studies consider that with force increasing, the CMC tends to shift to gamma-range (Omlor et al., 2007). For patients with different force levels, CMC is also significant in a certain frequency band. For example, in humans with cervical spinal cord injury, participants had an increased muscle co-activation associated with a decreased magnitude of the CMC in 10 Hz with antagonist muscles (Cremoux et al., 2017). Some authors proposed that lower limbs CMC was significantly reduced in SCA2 (spinocerebellar ataxia type 2) patients compared to healthy participants during repeated simultaneous flexion movements of fingers and wrist at a constant contraction level of 30% MVC (Velázquez-Pérez et al., 2017a).

Corticomuscular coherence is a key measurement to clarify the neural mechanism which is associated with an individual ability to stabilize muscle force output. CMC comparison between individuals with different force input level may provide a deeper understanding of the mechanisms. To sum up, no matter whether it is healthy subjects or patients, classification of force levels is one of the critical factors affecting CMC amplitude.

### Age Correlation

Aging is also associated with neuromuscular changes that can impair corticomuscular communication (Yoshida et al., 2017). These changes include decreasing in the recruited motor neurons (Kawamura et al., 1977a,b; Tomlinson and Irving, 1977) and the white matter volume of the posterior limbs of the internal capsule that contain the corticospinal tracts (Good et al., 2001; Salat et al., 2005). To be exact, previous studies have proposed age-related reduction in the amplitude of motor evoked potentials (i.e., corticospinal excitability) (Eisen et al., 1996) and CMC during sustained contractions of upper limb muscles (Graziadio et al., 2010; Bayram et al., 2015).

Age as one of the factors affects corticomuscular communication during movements should not be ignored. There was evidence for CMC in all age groups and lager, more distributed cortical networks in the children and elderly compared with young adults (Graziadio et al., 2010). James et al. (2008) compared the CMC among subjects in ages from infancy to elderly and showed prominent CMC differences during motor development in children compared to adults. CMC changed in functional connection with increasing force output helps to explain muscle weakness in elderly subjects. It has been reported that there was a strong link between cortical-muscular coherence and force output in the elderly individuals during abnormal walking (Clark et al., 2013). Bayram et al. (2015) investigated the functional CMC values in the elderly participants by calculating CMC during voluntary motor performance. The result showed that the CMC was significantly lower in older compared with young participants at different levels of elbow flexion force. Johnson and Shinohara (2012) investigated the differences of CMC between young and older adults during unilateral fine motor task, concurrent motor and cognitive tasks. They found that CMC was increasing in older adults with a significant influence of an additional cognitive task in alpha-range and young adults with greater beta-range CMC may exhibit more accurate motor than elderly adults. Besides, beta-range CMC in the motor cognitive task was negatively correlated with motor output error across young but not elderly adults. CMC changed in functional connection with increasing force output could help explain muscle weakness in elderly subjects.

Task dependency is a critical insight into the effects of aging on neural activity and motor performance. From previous studies (James et al., 2008; Graziadio et al., 2010), elderly subjects usually used simple unilateral tasks that required less awareness of attention. Beta-range CMC was suggested to be attenuated with reduced attention to a motor task (Kristeva-Feige et al., 2002; Johnson et al., 2011). The current research intends to examine the CMC in elderly adults in view of the importance of attention to tasks. For example, the alpha-band CMC on aging was increased with awareness on the task. That is because significant CMC was observed only during attention focusing or cognitive processing (Kristeva-Feige et al., 2002). Tasks that require distracting tasks reduce performance and are more common in elderly adults (Beauchet et al., 2005; Zijdewind et al., 2006; Voelcker-Rehage and Alberts, 2007; Hiraga et al., 2009). However, study also found a significant negative correlation between beta-range CMC and EMG variability across multiple trials which were observed within young adults rather than elderly adults (Graziadio et al., 2010).

The current research is mainly comparing CMC between young people and the elderly which employed unilateral tasks and relatively simple dual tasks. For instance, during unilateral task, beta-range CMC increased with aging from childhood (0 years old) to middle age (35 and 59 years old), but not to senior age (55–80 years old) (Graziadio et al., 2010). Alpha-band CMC during unilateral task was observed in elderly adults (55–80 years old) in more cases than in young people (21–35 years old) (Graziadio et al., 2010). By summarizing the significance of age in CMC on elderly people, we found that age has been gradually valued as a factor which could affect the magnitude of CMC. However, for functional significance of CMC, future study requires more awareness of attention to the comparison of CMC between young adults and elderly under the condition of bilateral complex tasks.

### Healthy Controls and Patients

To research the effects of corticomuscular coupling on motor injury and the possibility of clinical practice of CMC, some studies compare CMC between healthy subjects and patients with dyskinesia (i.e., stroke, Parkinson). Table 3 displays the comparison of healthy controls and patients of CMC. In terms of significant areas, the evaluation of CMC strength of healthy controls and patients provides evidence that corticomuscular coupling could apply in the rehabilitative evaluation of dyskinesia (Gao et al., 2017). CMC was implemented early in the Parkinson’s disease course which subsequent symptomatic relief with L-Dopa by CMC modulation (Salenius et al., 2002; Mckeown et al., 2006; Pollok et al., 2012). Usually, the CMC amplitude of patients on the affected side is lower than that of healthy subjects. For example, beta-range CMC was reduced dramatically in Amyotrophic Lateral Sclerosis (ALS) patients compared with healthy subjects during light voluntary muscular contraction (Proudfoot et al., 2018). Similarly, CMC was significantly lower in stroke patients compared with healthy participants for the anterior deltoid and brachii muscles at both beta (20–30 Hz) and lower gamma (30–40 Hz) ranges during the movement (Fang et al., 2009). Mima et al. (2001) and Fang et al. (2009) pointed that the functional coupling between cortex commands and corresponding muscular activities of stroke subjects was weaker than healthy subjects. Riquelme et al. (2014) investigated CMC during planning and execution of isotonic contractions in cerebral palsy (CP) patients and healthy subjects. The result showed that CP patients group displayed longer EMG onset latency and duration than healthy group and CMC in beta band of EEG was overall greater in CP than that in healthy controls. CMC in gamma-range was lower in CP group than healthy group, and brain functioning during movement initiation was altered in CP only at the beginning of muscular contraction. CMC is normally restored in patients with motor function recovery. One study reported that motor deficits secondary to acute stroke were attendant by a unilateral reduction in CMC (Nielsen et al., 2008), but the CMC magnitude was normalized with favorable functional recovery (Proudfoot et al., 2018). Research also demonstrated that the CMC strength was increasing with the restoration of motor function of the paretic limb. The measurement of CMC can reflect the recovery of motor function after stroke through quantifying interactions between the motor cortex and controlled muscle activities (Zheng et al., 2017).

**Table 3:** Health controls and patients of CMC.

Reference	Type	Sample	CMC result
Gao et al., 2017	EEG-EMGEMG-EEG	7 healthy controls5 stroke patients	Patients had lower CMC than healthy subjects
Velázquez-Pérez et al., 2017a	EEG-EMG	24 healthy controls19 SCA2 patients	Lower limbs CMC was significantly reduced in SCA2 patients as compared to healthy participants.
Sharifi et al., 2017	EEG-EMG	18 healthy controls18 essential tremor patients	CMC remained a relatively high level in healthy subjects. CMC level frequently dropped below the confidence level in patients.
Riquelme et al., 2014	EEG-EMG	15 healthy controls14 CP patients	CMC in gamma-band was lower in CP than in healthy controls
Proudfoot et al., 2018	EEG-EMG	17 healthy controls17 ALS patients	Beta-band CMC was significantly reduced in ALS patients compared to healthy controls.
Fang et al., 2009	EEG-EMG	8 healthy subjects21 stroke patients	Stroke patients had significantly lower CMC compared with healthy subjects for the anterior deltoid and brachii muscles.


*EMG, electromyography; EEG, electroencephalogram; SCA2, spinocerebellar ataxia type 2; CP, cerebral palsy; ALS, amyotrophic lateral sclerosis.*

Although relatively obvious differences on CMC analysis between patients and healthy individuals could be displayed in some studies, the consequences of CMC magnitude are varying. For instance, the significant CMC was only reported in a selection of patients (Hellwig et al., 2001), which indicated that the participation of the cortex in patients was not robust (Raethjen et al., 2007). Stroke patients manifest much more dispersive extent of cortical locations for peak CMC than healthy subjects, which purpose to keep with plastic reorganization of sensorimotor function (Rossiter et al., 2013; Farmer et al., 1993). In addition, it has been proved that CMC strength is modified in healthy subjects after immobilization (Lundbye-Jensen and Nielsen, 2008) or in neurological conditions such as essential (Muthuraman et al., 2010), neuropathic tremor (Weiss et al., 2010) and Parkinson disease (Weiss et al., 2012). Different choice of patients, analysis techniques and recording methods, types of sport duties, and possibly cognitive state (e.g., awareness of tremor) might be explained the reasons for inconsistent results (Sharifi et al., 2017). Through the comparative study of CMC between healthy controls and patients, we can find the potential clinical application of CMC, and the most direct application is motor rehabilitation. However, most of the current comparative studies could not give a quantitative index of CMC. There is only a simple comparison of CMC values between healthy controls and patients. If the CMC is to be clinically applied in the future, a more detailed classification of the affected CMC in patients with different movement disorders must be discussed and using CMC as a characteristic value to achieve a unified clinical measurement standard should also be studied.

## CMC Applications

As mentioned in the section of factors affecting CMC, the application and development trend of CMC should be the clinic rehabilitation of patients with sports disorders, even though most of the current CMC studies are still limited to the laboratory. The latest CMC studies focus on the types of patients, including stroke, Parkinson, tremor, etc. In the present review, a detailed overview of the current applications of CMC for patients with various motor disorders was provided.

### CMC Applications for Stroke Patients

For stroke patients at different stages, the performance of muscle contraction can be approximated as an indicator of stroke rehabilitation level. Therefore, it is extremely common to try CMC experiments in stroke patients. Table 4 shows the correlations of CMC and stroke. Normally, muscle atrophy in stroke patients cause a decrease in CMC. Some studies have also confirmed that stroke patients had dramatically lower CMC compared with healthy subjects for the anterior deltoid and brachii muscles (Fang et al., 2009). Similar result of restored CMC was also reported with well recovered patients with both Transcranial Magnetic Stimulation (TMS) and Magnetoencephalography (MEG) investigation (Braun et al., 2007). Rossiter et al. (2013) discovered that peak CMC in the contralesional hemisphere was found both in highly impaired patients and stroke patients with good functional recovery. This discovery provides evidence directly that brain regions in the contralesional hemisphere are participated in activities with the affected muscles in stroke patients. Zheng et al. (2017) demonstrated that the recovery level of motor function after stroke could be reflected by the measurement of CMC by quantifying interactions between the motor cortex and controlled muscle activities. Graziadio et al. (2012) proposed that the degree of global recovery after unilateral stroke in the chronic phase correlated with the degree symmetry achieved between the interdependent lesioned and non-lesioned corticospinal systems at CMC level. In addition to evaluation as rehabilitation indicator during the recovery of the stroke, CMC was also applied to distinguished types of stroke patients. Study investigated CMC in the chronic and acute stroke through following up the recovery courses, the results indicated CMC amplitude was increased on the unaffected side and CMC frequency was decreased on the affected side in acute stroke, however, there was no inter-hemispheric difference in CMC parameters of the chronic stroke. The dynamical changes of interaction between cortical cortex and muscle both at acute and chronic stage of stroke may be a characteristic parameter for clinical application of CMC.

**Table 4:** Correlations of CMC and Stroke.

Reference	Number of Patients	Stroke Type	CMC value (Patients vs Controls)
Zheng et al., 2017	1	Hemorrhage	Peak CMC in Beta Band (only patients)
Fang et al., 2009	21	17/21 Ischemia 4/21 Hemorrhage	Patients < Controls
Gao et al., 2017	5	1/5 Ischemia 4/5 Hemorrhage	Patients > Controls
Rossiter et al., 2013	25		Patients < Controls
von Carlowitz-Ghori et al., 2014	11	Ischemia	Patients < Controls
Mima et al., 2001	6		Patients < Controls
Larsen et al., 2017	19	Ischemia	Patients < Controls
Pan et al., 2018	12		ES CMC > sham ES CMC (only patients)
Chen et al., 2018	8	5/8 Ischemia 3/8 Hemorrhage	Patients < Controls
Belardinelli et al., 2017	8	3/8 Ischemia 5/8 Hemorrhage	Peak CMC in Beta Band (only patients)


*ES, electrical stimulation.*

### CMC Applications for Parkinson Patients

Parkinson’s disease (PD) is related to pathologically alter oscillatory activity (Krause et al., 2013). Table 5 shows the correlations of CMC and Parkinson disease. CMC as a neuro-physiological indicator of functional coupling between the primary motor cortex (M1) and peripheral muscles (Hari and Salenius, 1999; Krause et al., 2013) was applied as an index for PD symptoms variation early. Airaksinen et al. (2015b) found CMC decreased when they investigated defective cortical drive to muscle in PD. Similarly, CMC is a therapeutic indicator, PD patients had anomalously weak CMC after levodopa treatment during isometric contraction (Krause et al., 2013). In the recent Parkinson study, deep brain stimulation (DBS) of the subthalamic nucleus (STN) has the effects of improving motor symptoms and normalizing pathologically altered oscillations and applied to trace the rehabilitation of Parkinson patients with CMC. For example, STN-DBS was increasing the CMC amplitude of 10–30 Hz range for the tremorous hand because of the improvement of tremor by DBS (Park et al., 2009). Similar to this result, a slight increase of CMC during DBS was observed in eight patients on the average of 8 days studied after DBS implantation (Weiss et al., 2012). In addition, Airaksinen et al. (2015b) also showed DBS improved the CMC in advanced PD with large interindividual variability. Despite the differences in research results, it can be considered that CMC may be associated with the therapeutic effects of DBS. Similar to DBS, transcranial alternating current stimulation (tACS) can modulate cortical brain activity, some researchers were combined with tACS to study the CMC of PD patients. Study showed that decreased beta-range CMC and variability of fast lateral movements were due to motor cortex tACS at 20 Hz in PD patients (Krause et al., 2013).

**Table 5:** Correlations of CMC and Parkinson.

Reference	Number of Patients	Stimulation	CMC value (Patients vs Controls)
Airaksinen et al., 2015b	19	yes	DBS modifies patients’ CMC (only patients)
Krause et al., 2013	10	yes	Patients < Controls
Yoshida et al., 2017	10	no	Patients < Controls
Pollok et al., 2012	20	no	Patients < Controls


*DBS, deep brain stimulation.*

### CMC Applications for Tremor and Other Patients

In addition to stroke and Parkinson diseases, CMC may be possible regarded as an index to value some other movement disorders. Table 6 shows the correlations of CMC and other diseases. Tremor is one of the most common disorders. To confirm the motor cortex involved in essential tremor and factors that affect CMC strength, Sharifi et al. (2017) collected 18 essential tremor patients and the result showed that essential tremor CMC is desultory and subject to different functional duties. This result may serve to standardize tremor classification and the explanation of the analysis in clinical research. Proudfoot et al. (2018) aimed to measure pathological alteration to CMC resulting from ALC during steady force production. During light voluntary muscular contraction, beta-range CMC was dramatically reduced in ALS patients and propagation of motoric rhythms across the cortical cortex was also impaired. Velázquez-Pérez et al. (2017b) purposed to assess dysfunction of the corticospinal tract in spinocerebellar ataxia type 2 (SCA2) using CMC. Significant reductions of CMC in SCA2 patients showed an evidence of corticospinal tract dysfunction. The abnormal CMC could only be detected in lower limbs experiments rather than upper limbs experiments may result from the corticospinal tract length on the chrono dispersion of action potential conduction. Cerebral palsy (CP) is a motor impairment which could affect the muscular contractions and neural connections between motor cortex and relative muscle. Many researches indicated CP influenced the normal muscular activities such as reduced voluntary-contraction force (Barber et al., 2012; Braendvik and Roeleveld, 2012; De et al., 2012). Riquelme et al. (2014) compared CMC during planning and execution of hand movements in CP patients and healthy subjects. From the results, CP patients were characterized by an altered functional coupling through CMC analysis and CMC may consider as a tool for exploring deficits during early brain damage.

**Table 6:** Correlations of CMC and other diseases.

Reference	Number of Patients	Disease Type	CMC value (Patients vs Controls)
Sharifi et al., 2017	18	ET	Patients < Controls
Raethjen et al., 2013	37	ET	Patients > Controls
Hellwig et al., 2001	10	7/10 ET 3/10 EPT	Significant CMC at the tremor frequency in ET patients (only patients)
Velázquez-Pérez et al., 2017b	19	SCA2	Patients < Controls
Velázquez-Pérez et al., 2017a	15	SCA2	Patients < Controls
Proudfoot et al., 2018	17	ALS	Patients < Controls
Cremoux et al., 2017	8	SCI	Patients < Controls
Bravo-Esteban et al., 2014	14	SCI	Patients < Controls


*ET, essential tremor; EPT, enhanced physiological tremor; SCA2, spinocerebellar ataxia type 2; ALS, amyotrophic lateral sclerosis; SCI, spinal cord injury.*

**FIGURE 3:**
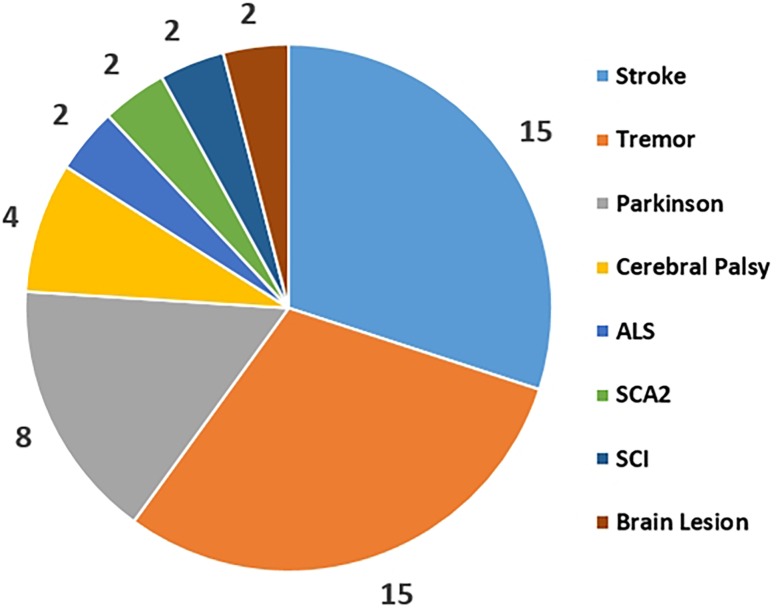
Corticomuscular coherence application areas distribution for the disorders.

Current literatures show that the applications of CMC in patients are simply regarded as a pathological indicator and are not clearly defined as clinically reliable parameters. The statistical comparison of different disorders use for the CMC study was collected form the published CMC papers as Figure 3 shown. This reflects that Stroke, Parkinson and Tremor are the top three diseases that be used to study CMC and explore the physiological variations during patients’ rehabilitation process. Rehabilitation diagnosis and treatment system based on CMC is the direction of future research. In addition, the design of CMC paradigm for patients with different diseases needs careful consideration.

## Discussion

The aim of this study is to explore the impacts of corticomuscular coherence and how these impacts affect the cortex-muscle coherence. The experimental paradigms almost include all parts of human body to study whether the coupling strength between the relevant cortex and muscles would provide potential values and contributions. In this research, the findings appropriately display the CMC in diverse parts have the effects on recovery monitoring, motion changing and otherwise. Experimental protocol is a significant cause to influence the results of CMC. Muscle contraction forms and muscle fatigue are the most important limit conditions during experiments (Siemionow et al., 2010; Ushiyama et al., 2010, 2011b; Bayraktaroglu et al., 2011). At the same time, similar oscillations may be detected from adjacent muscles result in motion decoding confusion, thus discrimination of body parts in CMC has a potential research significance. Oscillatory activities in both cortex and muscles are commonly appeared in different band frequencies (alpha-band, beta-band and gamma-band) (Muthukumaraswamy, 2011; Schoffelen et al., 2011). Alpha-band and beta-band CMC contribute more to actual motor function, while the peak CMC is usually observed within beta-band in healthy subjects and within alpha-band in functional disorder patients (Caviness et al., 2006). Furthermore, in some findings, alpha-band CMC is related to precise control of movements like finger movements and for steady isometric or isotonic contractions, beta-band normally associates with these kind of movements (Omlor et al., 2011). In general, diverse band frequencies in CMC represent different modes of neural communication between cerebral cortex and spinal cord. Muscle activation is modulated by cortical activity which may result in voluntary contractions. Underlying the co-activation of antagonist and agonist muscles, reducing cortical influences on inhibiting antagonist muscles is supposed to increase the muscles co-activation. Maximum voluntary contractions (MVC) is a standard to limit force level during the experiments in common use. With the force level increasing, the magnitude of corticomuscular coherence seems to enhance and muscles co-activation would decrease and these situations commonly appear in beta-band frequency rather than other band frequencies. The law of aging in CMC is also found. As age increases, the CMC would decrease gradually. However, the studies of aging are less than other factors correspondingly. To compare the CMC between healthy subjects and patients, healthy subjects have a higher CMC level than patients on account of nervous transmission damage (Patino et al., 2008; Meng et al., 2009). Meanwhile, peak CMC for healthy subjects emerges in beta-band while for patients, that shows in alpha-band.

In the present study, researchers are inclined to emphasize variance of corticomuscular coherence underlying distinct force levels and frequency bands (alpha, beta and gamma bands). Basically, a majority of researches of CMC would be able to consider band effects especially beta band and the effects of different bands in CMC have been studied thoroughly. CMC researches currently aim to seek a relationship or a correlation between CMC and other responsible factors which may modulate the amplitude of CMC. The challenges in the future focus on a greater depth of understanding the relationship between cortical and muscular activities and the applications in rehabilitation field and clinical field. The motion information decoding underlying the in-depth study of functional mechanism and CMC is possible to detect voluntary hand movements and more accurate than EEG only classification (Lou et al., 2013). If CMC is able to become a standard of motion decoding, it will eventually help exploit a new rehabilitation protocol. In general, the variance in CMC possible is related to communication issue between motor cortex and relative muscles. For instance, patients of cerebral lesion commonly have lower and intermittent CMC compare to healthy subjects, so they could not present a desirable movement since the motor impairment affects muscular contractions. Therefore, the further study, such as force controls or task complexity in both upper and lower limbs, would provide a precise CMC modulation protocols and a large number of experimental data basis for neuromuscular disorders. Furthermore, in rehabilitation field, experimental data basis might be benefit to establish a more reasonable and advanced rehabilitation programs for paralyzed patients and the improvement of CMC analysis could also provide a monitoring during the rehabilitation process. On the contrast, CMC monitoring could not only be used in patients’ rehabilitation processing but also be applied in healthy inspection for the healthy person. If CMC has an abnormal change during a period, there may be a risk of corticospinal tract degeneration and a timely diagnosis is helpful in the prevention of such diseases.

Comparing with CMC as a physiological index, many other metrics have been used for disorder detection. EMG amplitude and EMG median power frequency are usually good indicators of fatigue in multiple sclerosis (Tomasevic et al., 2013). Functional neuroimaging techniques such as fMRI, TMS and PET have been used to assess neural correlates of motor impairment and recovery over the past decades. Rehme et al. (2012) discovered that patients with stroke showed more task-related brain activation in both the affected and the unaffected hemisphere from PET and fMRI assessments. Volz et al. (2015) combined TMS, MRI, and connectivity analyses to investigate corticospinal tract (CST) injury in patients. Cortical excitability and motor network were effective connectivity for hand function recovery in chronic stroke patients.

Bourguignon et al. (2015) proposed that corticokinematic coherence (CKC) to reflect coupling between magnetoencephalo-graphic (MEG) signals and hand kinematics. It provided a reliable tool to monitor proprioceptive input to the cortex (Bourguignon et al., 2015). Intermuscular coherence (IMC) could quantify the strength of the coupling between cortex and the muscles. It was related to CMC in the beta band (Kilner et al., 1999) and reduced in the acute phase after stroke (Larsen et al., 2017). Dal Maso et al. (2018) explored correlations between event-related desynchronization (ERD), functional connectivity (FC) and CMC and skill retention, and suggested that cardiovascular exercise initiates significant changes in FC and CMC during motor memory consolidation (van Wijk et al., 2012). These metrics could be used as indicators of physiological activities through various forms of measurement, more or less relevant to the CMC.

Functional coupling between the motor cortex and muscle activity usually occurs with a time delay, which reflects signal propagation time between the brain and the muscle and information interaction (Xu et al., 2017). Perfect coherence (without temporal lags) does not exist which is only an ideal hypothesis in theory since multiple features influence the delays estimated using corticomuscular coherence, such as extra delays caused by the motor unit action potential, the duration of the corticomotoneuronal excitatory postsynaptic potential (EPSP), and a phase advance produced by motoneuron properties (Williams and Baker, 2008). The variety of temporal lags is the limitation of CMC as a physiological index. Some authors improved CMC by estimating the delay time. Govindan et al. (2005) used the method of maximizing coherence to obtain the time delay between two signals that were suitable for time delay estimation of narrow band coherence signals. Xu et al. (2017) proposed a CMC with time lag (CMCTL) function, which was the coherence displaced from a central observation point between segments of motor cortex EEG and EMG signals, and showed that it enhanced the CMC level and provided a more depth information on the temporal structure of CMC interaction than traditional CMC.

In summary, CMC research is still in a relatively early stage. Further exploration is needed in application, not only in rehabilitation and clinic for patients, but also in development of physical mechanism for healthy subjects.

## Conclusion

Corticomuscular coherence is a method to evaluate the coherence ability between motor cortex and muscles. For a more comprehensive understanding of the mechanism of CMC, the comparison between related factors shows that the peak CMC amplitude has a great probability to emerge under relatively high force level, beta frequency band. As age increases, CMC decreases under various degrees, which is also in line with the natural trend of muscle aging. The amplitude of CMC in healthy subjects is higher than that in patients in most cases. However, with the recovery of motor function of patients, CMC levels usually return to normal condition.

Current applications of CMC in patients is simply regarded as a pathological indicator and is not clearly defined as clinically reliable parameters. Further investigation is needed for a more complete understanding of enhancing CMC. Considering the use of different forms of muscle contraction to achieve superior results, scientific and reasonable paradigms are arranged to realize the target. Meanwhile, accurate classification of CMC on the affected side is needed to make CMC as an indicator in clinical application.

## Author Contributions

JL and YS wrote the body content and reviewed the whole article. HL reviewed the whole article and decided the final version.

## Conflict of Interest Statement

The authors declare that the research was conducted in the absence of any commercial or financial relationships that could be construed as a potential conflict of interest.
